# *mer*-Bis[2-(1,3-benzothiazol-2-yl)phenyl-κ^2^ *C* ^1^,*N*][3-phenyl-5-(2-pyridyl)-1,2,4-triazol-1-ido-κ^2^ *N* ^1^,*N* ^5^]iridium(III) deuterochloro­form 3.5-solvate

**DOI:** 10.1107/S1600536810031624

**Published:** 2010-08-11

**Authors:** Peter G. Jones, Andreas Freund, Andreas Weinkauf, Wolfgang Kowalsky, Hans-Hermann Johannes

**Affiliations:** a Institut für Anorganische und Analytische Chemie, Technical University of Braunschweig, Postfach 3329, 38023 Braunschweig, Germany; b Labor für Elektrooptik am Institut für Hochfrequenztechnik, Technical University of Braunschweig, Postfach 3329, 38023 Braunschweig, Germany

## Abstract

In the title compound, [Ir(C_13_H_9_N_4_)(C_13_H_8_NS)_2_]·3.5CDCl_3_, the coordination at iridium is octa­hedral, but with narrow ligand bite angles. The bond lengths at iridium show the expected *trans* influence, with the Ir—N bonds *trans* to C being appreciably longer than those *trans* to N. The chelate rings are mutually perpendicular, the inter­planar angles between them all lying within 6° of 90°. All ligands are approximately planar; the maximum inter­planar angles within ligands are *ca* 10°. The three ordered deuterochloro­form mol­ecules are all involved in C⋯D—*A* contacts that can be inter­preted as hydrogen bonds of various types. The fourth deuterochloroform is disordered over an inversion centre.

## Related literature

For the preparation of iridium complexes, see: Lamansky *et al.* (2001 ▶); Tamayo *et al.* (2003 ▶). For the photoluminescent properties and color tuning of cyclo­metalated iridium complexes, see: Grushin *et al.* (2001 ▶); Kwon *et al.* (2005 ▶); You & Park (2005 ▶). For general background to organic light-emitting diodes (OLEDs), see: Holder *et al.* (2005 ▶); Kappaun *et al.* (2008 ▶). For a related recent publication from our groups, see: Jones *et al.* (2010 ▶).
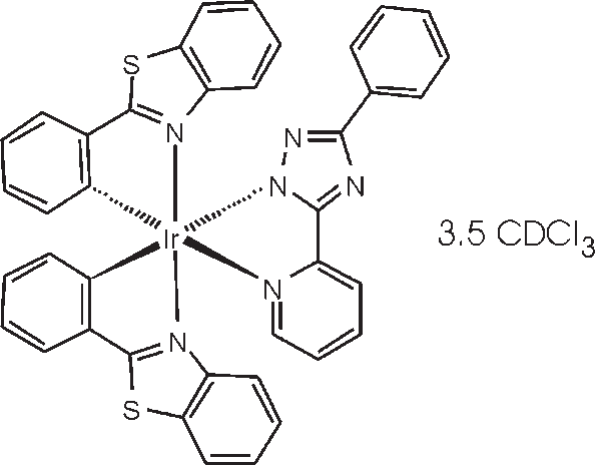


## Experimental

### 

#### Crystal data

[Ir(C_13_H_9_N_4_)(C_13_H_8_NS)_2_]·3.5CDCl_3_
*M* *_r_* = 1254.78
Triclinic, 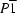

*a* = 11.7521 (4) Å
*b* = 13.5592 (4) Å
*c* = 15.8373 (4) Å
α = 74.045 (3)°
β = 79.247 (3)°
γ = 76.002 (3)°
*V* = 2334.80 (12) Å^3^
*Z* = 2
Mo *K*α radiation
μ = 3.59 mm^−1^
*T* = 100 K
0.20 × 0.10 × 0.08 mm

#### Data collection

Oxford Diffraction Xcalibur, Eos diffractometer
Absorption correction: multi-scan (*CrysAlis PRO*; Oxford Diffraction, 2010 ▶). *T*_min_ = 0.902, *T*_max_ = 1.000
94997 measured reflections
13407 independent reflections
10433 reflections with *I* > 2σ(*I*)
*R*_int_ = 0.058

#### Refinement

*R*[*F* ^2^ > 2σ(*F* ^2^)] = 0.027
*wR*(*F* ^2^) = 0.047
*S* = 0.88
13407 reflections
572 parameters
6 restraints
H-atom parameters constrained
Δρ_max_ = 1.88 e Å^−3^
Δρ_min_ = −1.70 e Å^−3^

### 

Data collection: *CrysAlis PRO* (Oxford Diffraction, 2010 ▶); cell refinement: *CrysAlis PRO*; data reduction: *CrysAlis PRO*; program(s) used to solve structure: *SHELXS97* (Sheldrick, 2008 ▶); program(s) used to refine structure: *SHELXL97* (Sheldrick, 2008 ▶); molecular graphics: *XP* (Siemens, 1994 ▶); software used to prepare material for publication: *SHELXL97*.

## Supplementary Material

Crystal structure: contains datablocks I, global. DOI: 10.1107/S1600536810031624/bt5321sup1.cif

Structure factors: contains datablocks I. DOI: 10.1107/S1600536810031624/bt5321Isup2.hkl

Additional supplementary materials:  crystallographic information; 3D view; checkCIF report

## Figures and Tables

**Table 1:** Hydrogen-bond geometry (Å, °) *Cg* is the centroid of the C27–C32 ring.

*D*—H⋯*A*	*D*—H	H⋯*A*	*D*⋯*A*	*D*—H⋯*A*
C99—D99⋯N19	1.00	2.18	3.180 (4)	175
C98—D98⋯S3′	1.00	3.04	3.660 (3)	121
C97—D97⋯*Cg*	1.00	2.50	3.50	173

**Fig. 1.**
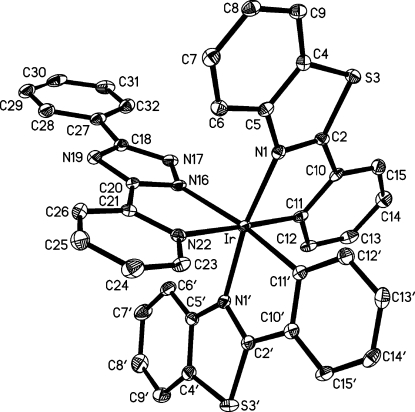
Structure of the title compound in the crystal. Ellipsoids represent 50% probability levels. Solvent molecules and hydrogen atoms are omitted for clarity.

## supplementary crystallographic information

### Comment

Organometallic phosphorescent materials based on iridium(III) play an important role in the field of organic light-emitting diodes (OLEDs). They exhibit high quantum efficiencies, relatively short phosphorescent lifetimes, and facile colour tuning by modification of the ligand structures. Moreover, this class of materials can lead to OLEDs with 100% internal quantum efficiencies by harvesting both singlet and triplet excitons. In this contribution, we have synthesized and characterized a new iridium(III) complex with two 2-phenylbenzothiazoles as chromophoric ligands and 3-phenyl-5-(2-pyridine)-1,2,4-triazole as ancillary ligand, and report here the crystal structure of this compound.

The structure of the title complex is shown in Fig. 1. It crystallizes with four molecules of deuterochloroform, one of which is disordered cleanly over an inversion centre. The coordination at iridium is octahedral, with the major deviations in angles arising from the restricted bite of the chelating ligands: N1—Ir—C11 79.54 (9), N1'—Ir—C11' 79.98 (9), N16—Ir—N22 76.03 (8)°. The bond lengths at iridium show the expected *trans* influence, with Ir—N16 and Ir—N22, 2.126 (2) and 2.160 (2) Å respectively, *trans* to C being appreciably longer than the mutually *trans* Ir—N1 2.060 (2) and Ir—N1' 2.064 (2) Å. The interplanar angles between the chelate rings all lie within 6° of 90°. Within the ligands, the interplanar angles between phenyl and benzothiazole are 9.9 (1) and 10.8 (1)°, whereas in the triazole ligand the pyridyl and phenyl rings subtend angles of 0.4 (2) and 9.2 (2)° respectively to the triazole ring.

The three ordered deuterochloroform molecules are all involved in C···D—A contacts that can be interpreted as hydrogen bonds of various types (Table 1); D99···N19 2.18, D98···S3' 3.04 (but with a narrow angle of 121°), D97···*Cg*(C27–C32) 2.50 Å.

### Experimental

A mixture of bis(2-phenylbenzo[*d*]thiazole)-iridium(III)-µ-chloro bridged dimer (300 mg, 231 µmol), 3-phenyl-5-(2-pyridyl)-1,2,4-triazole (129 mg, 579 µmol) and potassium *tert*-butoxide (65 mg, 579 µmol) in dry dichloromethane (10 ml) and dry ethanol (3 ml) was stirred overnight at room temperature under nitrogen atmosphere. The solvent was then removed under reduced pressure and the residue was purified *via* flash-chromatography on silica gel (eluent: dichloromethane/acetone = 10: 1, *R_f_* = 0.57) to yield an orange solid (299 mg, 77%). m.p. 306 °C.

- ^1^H NMR (CDCl_3_, 600 MHz): δ = 8.22 (d, *J* = 7.9 Hz, 1H), 8.13–8.11 (m, 2H), 7.83 (ddd, *J* = 5.5, 1.5, 0.9 Hz, 1H), 7.80 (ddd, *J* = 7.8, 7.8, 1.6 Hz, 1H), 7.78–7.77 (m, 1H), 7.75–7.73 (m, 3H), 7.34–7.31 (m, 2H), 7.27–7.23 (m, 2H), 7.21 (ddd, *J* = 8.2, 7.3, 1.0 Hz, 1H), 7.13–7.10 (m, 2H), 7.02 (ddd, *J* = 7.5, 7.5, 1.1 Hz, 1H), 6.99 (ddd, *J* = 7.5, 7.5, 1.1 Hz, 1H), 6.97 (d, *J* = 8.4 Hz, 1H), 6.93 (ddd, *J* = 8.5, 7.3, 1.2 Hz, 1H), 6.83 (ddd, *J* = 7.5, 7.5, 1.3 Hz, 1H), 6.80 (ddd, *J* = 7.5, 7.5, 1.4 Hz, 1H), 6.54 (d, *J* = 7.4 Hz, 1H), 6.48 (dd, *J* = 7.7, 0.4 Hz, 1H), 6.23 (d, J = 8.4 Hz, 1H) p.p.m..

- ^13^C NMR (CDCl_3_, 150 MHz): δ = 180.96, 180.35, 165.06, 163.63, 154.92, 152.95, 149.80, 149.75, 149.71, 149.60, 141.22, 140.77, 138.27, 134.18, 133.52, 132.93, 131.43, 131.37, 130.93, 130.83, 128.32, 128.21, 127.66, 127.02, 126.20, 126.04, 125.94, 125.46, 124.89, 123.84, 122.94, 122.15, 122.10, 121.73, 121.16, 120.36, 118.04 p.p.m..

- EI—MS: m/*z* (%) = 834 (100) [*M*^+^], 756 (9), 613 (35), 286 (12), 211 (41).

- IR: ν-tilde = 3055 (w), 1606 (w), 1581 (w), 1436 (*m*), 1408 (*m*), 1322 (w), 1297 (w), 1266 (*m*), 1157 (w), 1024 (*m*), 993 (*m*), 791 (w), 752 (*s*), 723 (*versus*), 696 (*m*), 582 (w) cm^-1^.

- UV/Vis (CH_2_Cl_2_): λ (ε [cm^-1^*M*^-1^]) = 228 (47000), 272 (45500), 310 (36600), 323 (35600), 350 (15400), 383 (9400), 454 (4200) nm.

- Elemental analysis: calculated for C_39_H_25_IrN_6_S_2_: C 56.16, H 3.02, N 10.08, S 7.69%; found: C 56.29, H 3.02, N 10.39 S 7.47%.

Single crystals were obtained by evaporation from CDCl_3_ in an NMR tube.

### Refinement

Hydrogen atoms were included at calculated positions using a riding model with aromatic C—H 0.95, *sp*^3^-C—H 1.00 Å. The *U*(H) values were fixed at 1.2 × *U*_eq_(C) of the parent C atom.

The chloroform molecule C96–Cl12 is disordered over an inversion centre; the carbon was refined isotropically. Distance restraints were employed to improve refinement stability.

There are several peaks of 1.1–1.9 e Å^-3^ either *ca* 0.9 Å from the Ir atom, which may reasonably be attributed to residual absorption errors, or in the solvent region, corresponding to slight extra disorder or irregular displacement features.

### Crystal data

**Table:**

[Ir(C_13_H_9_N_4_)(C_13_H_8_NS)_2_]·3.5CDCl_3_	*Z* = 2
*M**_r_* = 1254.78	*F*(000) = 1226
Triclinic, *P*1	*D*_x_ = 1.785 Mg m^−^^3^
*a* = 11.7521 (4) Å	Mo *K*α radiation, λ = 0.71073 Å
*b* = 13.5592 (4) Å	Cell parameters from 31145 reflections
*c* = 15.8373 (4) Å	θ = 2.2–30.8°
α = 74.045 (3)°	µ = 3.59 mm^−^^1^
β = 79.247 (3)°	*T* = 100 K
γ = 76.002 (3)°	Prism, orange
*V* = 2334.80 (12) Å^3^	0.20 × 0.10 × 0.08 mm

### Data collection

**Table:**

Oxford Diffraction Xcalibur, Eos diffractometer	13407 independent reflections
Radiation source: fine-focus sealed tube	10433 reflections with *I* > 2σ(*I*)
graphite	*R*_int_ = 0.058
Detector resolution: 16.1419 pixels mm^-1^	θ_max_ = 30.0°, θ_min_ = 2.2°
ω–scan	*h* = −16→16
Absorption correction: multi-scan *CrysAlis PRO*, Oxford Diffraction (2010).	*k* = −19→18
*T*_min_ = 0.902, *T*_max_ = 1.000	*l* = −22→22
94997 measured reflections	

### Refinement

**Table:**

Refinement on *F*^2^	Primary atom site location: structure-invariant direct methods
Least-squares matrix: full	Secondary atom site location: difference Fourier map
*R*[*F*^2^ > 2σ(*F*^2^)] = 0.027	Hydrogen site location: inferred from neighbouring sites
*wR*(*F*^2^) = 0.047	H-atom parameters constrained
*S* = 0.88	*w* = 1/[σ^2^(*F*_o_^2^) + (0.0175*P*)^2^] where *P* = (*F*_o_^2^ + 2*F*_c_^2^)/3
13407 reflections	(Δ/σ)_max_ = 0.002
572 parameters	Δρ_max_ = 1.88 e Å^−^^3^
6 restraints	Δρ_min_ = −1.70 e Å^−^^3^

### Special details

**Table:**

Geometry. All e.s.d.'s (except the e.s.d. in the dihedral angle between two l.s. planes) are estimated using the full covariance matrix. The cell e.s.d.'s are taken into account individually in the estimation of e.s.d.'s in distances, angles and torsion angles; correlations between e.s.d.'s in cell parameters are only used when they are defined by crystal symmetry. An approximate (isotropic) treatment of cell e.s.d.'s is used for estimating e.s.d.'s involving l.s. planes.Least-squares planes (*x*,*y*,*z* in crystal coordinates) and deviations from them (* indicates atom used to define plane)7.9800 (0.0061) *x* - 1.7224 (0.0133) *y* + 10.9969 (0.0085) *z* = 7.6717 (0.0069)* -0.0488 (0.0009) Ir * 0.0413 (0.0012) N1 * -0.0066 (0.0014) C2 * -0.0540 (0.0015) C10 * 0.0680 (0.0013) C11Rms deviation of fitted atoms = 0.04838.3461 (0.0059) *x* + 9.9411 (0.0076) *y* - 1.7893 (0.0166) *z* = 2.3716 (0.0114)Angle to previous plane (with approximate e.s.d.) = 87.72 (0.06)* -0.0493 (0.0009) Ir * 0.0551 (0.0013) N1' * -0.0297 (0.0015) C2' * -0.0327 (0.0016) C10' * 0.0566 (0.0013) C11'Rms deviation of fitted atoms = 0.0461- 2.3439 (0.0116) *x* + 9.9572 (0.0069) *y* + 10.8389 (0.0095) *z* = 7.7705 (0.0077)Angle to previous plane (with approximate e.s.d.) = 84.16 (0.06)* -0.0054 (0.0009) Ir * 0.0091 (0.0014) N16 * -0.0083 (0.0016) C20 * 0.0006 (0.0016) C21 * 0.0041 (0.0013) N22Rms deviation of fitted atoms = 0.00637.9800 (0.0061) *x* - 1.7224 (0.0133) *y* + 10.9969 (0.0085) *z* = 7.6717 (0.0069)Angle to previous plane (with approximate e.s.d.) = 89.73 (0.07)* -0.0488 (0.0009) Ir * 0.0413 (0.0012) N1 * -0.0066 (0.0014) C2 * -0.0540 (0.0015) C10 * 0.0680 (0.0013) C11Rms deviation of fitted atoms = 0.04838.2788 (0.0045) *x* - 0.5241 (0.0062) *y* + 11.4855 (0.0070) *z* = 8.1544 (0.0022)Angle to previous plane (with approximate e.s.d.) = 5.08 (0.10)* 0.0042 (0.0016) N1 * 0.0078 (0.0016) C2 * -0.0070 (0.0011) S3 * -0.0048 (0.0020) C4 * -0.0050 (0.0020) C5 * -0.0049 (0.0018) C6 * 0.0005 (0.0018) C7 * 0.0043 (0.0018) C8 * 0.0049 (0.0017) C9Rms deviation of fitted atoms = 0.00528.4858 (0.0077) *x* - 2.4161 (0.0127) *y* + 9.6572 (0.0118) *z* = 7.0197 (0.0090)Angle to previous plane (with approximate e.s.d.) = 9.93 (0.11)* 0.0136 (0.0016) C10 * -0.0217 (0.0016) C11 * 0.0118 (0.0016) C12 * 0.0070 (0.0017) C13 * -0.0158 (0.0017) C14 * 0.0052 (0.0017) C15Rms deviation of fitted atoms = 0.01377.1925 (0.0056) *x* + 9.8261 (0.0068) *y* - 3.8421 (0.0083) *z* = 0.9988 (0.0081)Angle to previous plane (with approximate e.s.d.) = 85.34 (0.06)* -0.0364 (0.0018) N1' * 0.0610 (0.0017) C2' * 0.0364 (0.0013) S3' * -0.0552 (0.0023) C4' * -0.0535 (0.0022) C5' * 0.0058 (0.0021) C6' * 0.0512 (0.0022) C7' * 0.0250 (0.0022) C8' * -0.0342 (0.0021) C9'Rms deviation of fitted atoms = 0.04318.1235 (0.0081) *x* + 10.5535 (0.0086) *y* - 0.9454 (0.0159) *z* = 2.9872 (0.0090)Angle to previous plane (with approximate e.s.d.) = 10.83 (0.12)* 0.0050 (0.0017) C10' * 0.0034 (0.0017) C11' * -0.0084 (0.0018) C12' * 0.0049 (0.0018) C13' * 0.0036 (0.0018) C14' * -0.0084 (0.0017) C15'Rms deviation of fitted atoms = 0.0060- 2.5414 (0.0127) *x* + 9.7359 (0.0101) *y* + 10.9194 (0.0127) *z* = 7.8052 (0.0089)Angle to previous plane (with approximate e.s.d.) = 81.42 (0.07)* 0.0037 (0.0018) C21 * -0.0086 (0.0016) N22 * 0.0048 (0.0018) C23 * 0.0039 (0.0020) C24 * -0.0087 (0.0020) C25 * 0.0048 (0.0019) C26Rms deviation of fitted atoms = 0.0061- 2.5107 (0.0151) *x* + 9.8045 (0.0109) *y* + 10.8623 (0.0148) *z* = 7.7407 (0.0143)Angle to previous plane (with approximate e.s.d.) = 0.43 (0.17)* -0.0022 (0.0014) N16 * 0.0011 (0.0014) N17 * 0.0003 (0.0015) C18 * -0.0016 (0.0014) N19 * 0.0024 (0.0015) C20Rms deviation of fitted atoms = 0.0017- 2.4518 (0.0127) *x* + 10.9099 (0.0091) *y* + 9.0926 (0.0145) *z* = 6.2049 (0.0171)Angle to previous plane (with approximate e.s.d.) = 9.27 (0.16)* 0.0117 (0.0018) C27 * -0.0068 (0.0019) C28 * -0.0042 (0.0019) C29 * 0.0103 (0.0019) C30 * -0.0052 (0.0019) C31 * -0.0058 (0.0019) C32Rms deviation of fitted atoms = 0.0078
Refinement. Refinement of *F*^2^ against ALL reflections. The weighted *R*-factor *wR* and goodness of fit *S* are based on *F*^2^, conventional *R*-factors *R* are based on *F*, with *F* set to zero for negative *F*^2^. The threshold expression of *F*^2^ > σ(*F*^2^) is used only for calculating *R*-factors(gt) *etc*. and is not relevant to the choice of reflections for refinement. *R*-factors based on *F*^2^ are statistically about twice as large as those based on *F*, and *R*- factors based on ALL data will be even larger.

### Fractional atomic coordinates and isotropic or equivalent isotropic displacement parameters (Å^2^)

**Table:**

	*x*	*y*	*z*	*U*_iso_*/*U*_eq_	Occ. (<1)
Ir	0.185225 (9)	0.183852 (9)	0.587566 (7)	0.01036 (3)	
N1	0.29011 (17)	0.09344 (16)	0.50550 (13)	0.0113 (5)	
C2	0.3713 (2)	0.1390 (2)	0.44934 (16)	0.0113 (5)	
S3	0.46148 (5)	0.06657 (5)	0.37977 (4)	0.01364 (13)	
C4	0.3867 (2)	−0.0356 (2)	0.42917 (16)	0.0115 (5)	
C5	0.2971 (2)	−0.00772 (19)	0.49503 (16)	0.0115 (5)	
C6	0.2262 (2)	−0.0791 (2)	0.54288 (16)	0.0150 (6)	
H6	0.1647	−0.0615	0.5877	0.018*	
C7	0.2476 (2)	−0.1758 (2)	0.52351 (17)	0.0158 (6)	
H7	0.2001	−0.2250	0.5556	0.019*	
C8	0.3378 (2)	−0.2028 (2)	0.45764 (16)	0.0153 (6)	
H8	0.3504	−0.2698	0.4457	0.018*	
C9	0.4085 (2)	−0.1336 (2)	0.40982 (16)	0.0135 (5)	
H9	0.4701	−0.1519	0.3652	0.016*	
C10	0.3802 (2)	0.24172 (19)	0.45468 (16)	0.0118 (5)	
C11	0.3038 (2)	0.2743 (2)	0.52632 (16)	0.0120 (5)	
C12	0.3162 (2)	0.3678 (2)	0.54230 (17)	0.0145 (6)	
H12	0.2699	0.3914	0.5916	0.017*	
C13	0.3950 (2)	0.4261 (2)	0.48715 (18)	0.0179 (6)	
H13	0.4009	0.4895	0.4991	0.022*	
C14	0.4655 (2)	0.3945 (2)	0.41489 (18)	0.0181 (6)	
H14	0.5173	0.4366	0.3768	0.022*	
C15	0.4593 (2)	0.3009 (2)	0.39911 (17)	0.0163 (6)	
H15	0.5084	0.2770	0.3509	0.020*	
N16	0.26737 (17)	0.08428 (16)	0.69814 (13)	0.0120 (5)	
N17	0.36908 (17)	0.07191 (16)	0.73312 (13)	0.0133 (5)	
C18	0.3628 (2)	−0.0086 (2)	0.80427 (16)	0.0143 (5)	
N19	0.26461 (18)	−0.04920 (17)	0.81804 (13)	0.0151 (5)	
C20	0.2083 (2)	0.0125 (2)	0.74975 (16)	0.0125 (5)	
C21	0.0947 (2)	0.0096 (2)	0.72860 (16)	0.0138 (5)	
N22	0.06182 (18)	0.08334 (16)	0.65410 (13)	0.0129 (5)	
C23	−0.0419 (2)	0.0868 (2)	0.62811 (17)	0.0157 (6)	
H23	−0.0659	0.1387	0.5772	0.019*	
C24	−0.1149 (2)	0.0181 (2)	0.67229 (19)	0.0225 (6)	
H24	−0.1876	0.0227	0.6520	0.027*	
C25	−0.0811 (2)	−0.0577 (2)	0.74654 (19)	0.0221 (6)	
H25	−0.1295	−0.1066	0.7773	0.026*	
C26	0.0244 (2)	−0.0613 (2)	0.77559 (17)	0.0188 (6)	
H26	0.0483	−0.1119	0.8272	0.023*	
C27	0.4565 (2)	−0.0474 (2)	0.86363 (17)	0.0155 (6)	
C28	0.4390 (2)	−0.1198 (2)	0.94374 (17)	0.0192 (6)	
H28	0.3699	−0.1488	0.9584	0.023*	
C29	0.5226 (2)	−0.1498 (2)	1.00257 (18)	0.0241 (7)	
H29	0.5103	−0.1990	1.0574	0.029*	
C30	0.6229 (2)	−0.1082 (2)	0.98128 (18)	0.0230 (7)	
H30	0.6790	−0.1277	1.0221	0.028*	
C31	0.6426 (2)	−0.0383 (2)	0.90106 (18)	0.0216 (6)	
H31	0.7130	−0.0111	0.8863	0.026*	
C32	0.5599 (2)	−0.0074 (2)	0.84167 (17)	0.0179 (6)	
H32	0.5737	0.0406	0.7864	0.021*	
N1'	0.08049 (17)	0.29382 (16)	0.65163 (13)	0.0123 (5)	
C2'	−0.0090 (2)	0.35294 (19)	0.60986 (16)	0.0125 (5)	
S3'	−0.10350 (6)	0.44093 (5)	0.66450 (4)	0.01738 (14)	
C4'	−0.0142 (2)	0.3993 (2)	0.74905 (17)	0.0155 (6)	
C5'	0.0825 (2)	0.3214 (2)	0.73020 (16)	0.0132 (5)	
C6'	0.1713 (2)	0.2840 (2)	0.78538 (17)	0.0182 (6)	
H6'	0.2378	0.2315	0.7734	0.022*	
C7'	0.1597 (2)	0.3254 (2)	0.85796 (18)	0.0232 (7)	
H7'	0.2198	0.3013	0.8958	0.028*	
C8'	0.0621 (3)	0.4016 (2)	0.87686 (18)	0.0243 (7)	
H8'	0.0564	0.4279	0.9275	0.029*	
C9'	−0.0265 (2)	0.4394 (2)	0.82311 (17)	0.0219 (6)	
H9'	−0.0935	0.4909	0.8361	0.026*	
C10'	−0.0148 (2)	0.34173 (19)	0.52221 (16)	0.0124 (5)	
C11'	0.0796 (2)	0.26639 (19)	0.49410 (16)	0.0111 (5)	
C12'	0.0820 (2)	0.2557 (2)	0.40818 (17)	0.0163 (6)	
H12'	0.1427	0.2053	0.3860	0.020*	
C13'	−0.0031 (2)	0.3177 (2)	0.35466 (17)	0.0179 (6)	
H13'	0.0018	0.3097	0.2963	0.021*	
C14'	−0.0948 (2)	0.3908 (2)	0.38492 (17)	0.0167 (6)	
H14'	−0.1522	0.4324	0.3477	0.020*	
C15'	−0.1017 (2)	0.4026 (2)	0.46962 (17)	0.0160 (6)	
H15'	−0.1646	0.4514	0.4918	0.019*	
C97	0.7155 (2)	−0.3134 (2)	0.88203 (17)	0.0221 (6)	
D97	0.6606	−0.2466	0.8897	0.026*	
Cl1	0.67186 (6)	−0.41646 (6)	0.96693 (5)	0.02871 (17)	
Cl2	0.70766 (6)	−0.32965 (6)	0.77698 (4)	0.02763 (17)	
Cl3	0.86049 (6)	−0.30466 (6)	0.89109 (5)	0.02593 (16)	
C98	−0.3809 (3)	0.3533 (3)	0.7609 (2)	0.0494 (11)	
D98	−0.3574	0.4101	0.7099	0.059*	
Cl4	−0.52591 (11)	0.34664 (11)	0.75553 (8)	0.1027 (6)	
Cl5	−0.28516 (11)	0.23563 (9)	0.75073 (7)	0.0721 (3)	
Cl6	−0.36850 (8)	0.38391 (8)	0.85894 (7)	0.0564 (3)	
C99	0.2676 (2)	−0.2928 (2)	0.85817 (19)	0.0258 (7)	
D99	0.2719	−0.2173	0.8456	0.031*	
Cl7	0.23359 (9)	−0.31505 (9)	0.76311 (6)	0.0599 (3)	
Cl8	0.40549 (7)	−0.36806 (7)	0.88483 (6)	0.0412 (2)	
Cl9	0.15616 (7)	−0.31749 (7)	0.94744 (6)	0.0427 (2)	
C96	0.9372 (5)	0.0481 (5)	1.0020 (4)	0.0311 (15)*	0.50
H96	0.8893	0.0731	1.0541	0.037*	0.50
Cl10	1.0842 (4)	0.0597 (5)	0.9970 (5)	0.0593 (16)	0.50
Cl11	0.88004 (16)	0.12749 (14)	0.90568 (11)	0.0413 (4)	0.50
Cl12	0.9281 (5)	−0.0812 (5)	1.0147 (5)	0.0587 (15)	0.50

### Atomic displacement parameters (Å^2^)

**Table:**

	*U*^11^	*U*^22^	*U*^33^	*U*^12^	*U*^13^	*U*^23^
Ir	0.00954 (5)	0.00903 (5)	0.01150 (5)	−0.00029 (4)	−0.00206 (3)	−0.00174 (3)
N1	0.0096 (11)	0.0124 (12)	0.0118 (11)	−0.0018 (9)	−0.0041 (8)	−0.0014 (9)
C2	0.0079 (12)	0.0131 (14)	0.0127 (12)	0.0000 (10)	−0.0030 (10)	−0.0035 (10)
S3	0.0124 (3)	0.0138 (3)	0.0148 (3)	−0.0027 (3)	0.0001 (2)	−0.0048 (3)
C4	0.0093 (13)	0.0142 (14)	0.0118 (13)	−0.0036 (11)	−0.0032 (10)	−0.0022 (11)
C5	0.0106 (12)	0.0110 (13)	0.0140 (13)	−0.0014 (10)	−0.0058 (10)	−0.0026 (10)
C6	0.0126 (13)	0.0160 (15)	0.0157 (13)	−0.0022 (11)	−0.0009 (10)	−0.0039 (11)
C7	0.0161 (14)	0.0141 (14)	0.0183 (14)	−0.0055 (11)	−0.0026 (11)	−0.0031 (11)
C8	0.0175 (14)	0.0126 (14)	0.0171 (14)	−0.0005 (11)	−0.0064 (11)	−0.0052 (11)
C9	0.0102 (13)	0.0142 (14)	0.0162 (13)	−0.0002 (11)	−0.0041 (10)	−0.0043 (11)
C10	0.0113 (12)	0.0094 (13)	0.0141 (13)	−0.0006 (10)	−0.0062 (10)	−0.0003 (10)
C11	0.0102 (13)	0.0113 (14)	0.0145 (13)	0.0015 (10)	−0.0062 (10)	−0.0034 (11)
C12	0.0111 (13)	0.0120 (14)	0.0198 (14)	0.0023 (11)	−0.0056 (11)	−0.0046 (11)
C13	0.0152 (14)	0.0124 (14)	0.0278 (16)	−0.0010 (11)	−0.0079 (12)	−0.0056 (12)
C14	0.0139 (14)	0.0172 (15)	0.0229 (15)	−0.0078 (12)	−0.0013 (11)	−0.0008 (12)
C15	0.0121 (13)	0.0195 (15)	0.0151 (13)	−0.0030 (11)	−0.0013 (10)	−0.0010 (11)
N16	0.0091 (10)	0.0112 (12)	0.0142 (11)	0.0020 (9)	−0.0030 (8)	−0.0033 (9)
N17	0.0112 (11)	0.0144 (12)	0.0144 (11)	0.0015 (9)	−0.0033 (9)	−0.0062 (9)
C18	0.0166 (13)	0.0122 (14)	0.0125 (13)	0.0016 (11)	−0.0018 (10)	−0.0043 (11)
N19	0.0159 (11)	0.0134 (12)	0.0132 (11)	0.0006 (9)	−0.0023 (9)	−0.0016 (9)
C20	0.0129 (13)	0.0098 (13)	0.0122 (13)	0.0011 (10)	−0.0004 (10)	−0.0020 (10)
C21	0.0124 (13)	0.0122 (14)	0.0149 (13)	0.0008 (11)	0.0005 (10)	−0.0047 (11)
N22	0.0123 (11)	0.0110 (12)	0.0148 (11)	−0.0015 (9)	0.0006 (9)	−0.0044 (9)
C23	0.0133 (13)	0.0137 (14)	0.0188 (14)	0.0011 (11)	−0.0052 (11)	−0.0031 (11)
C24	0.0134 (14)	0.0198 (16)	0.0331 (17)	−0.0036 (12)	−0.0044 (12)	−0.0033 (13)
C25	0.0159 (14)	0.0174 (16)	0.0298 (17)	−0.0075 (12)	0.0031 (12)	−0.0011 (13)
C26	0.0180 (14)	0.0153 (15)	0.0178 (14)	−0.0011 (12)	0.0002 (11)	0.0014 (11)
C27	0.0200 (14)	0.0130 (14)	0.0141 (13)	0.0022 (11)	−0.0057 (11)	−0.0068 (11)
C28	0.0217 (15)	0.0174 (15)	0.0163 (14)	0.0026 (12)	−0.0047 (11)	−0.0045 (12)
C29	0.0315 (17)	0.0180 (16)	0.0161 (14)	0.0074 (13)	−0.0057 (12)	−0.0021 (12)
C30	0.0261 (16)	0.0193 (16)	0.0231 (15)	0.0105 (13)	−0.0155 (13)	−0.0092 (13)
C31	0.0182 (14)	0.0223 (17)	0.0263 (16)	0.0028 (12)	−0.0081 (12)	−0.0115 (13)
C32	0.0222 (15)	0.0150 (15)	0.0167 (14)	−0.0003 (12)	−0.0067 (11)	−0.0043 (11)
N1'	0.0127 (11)	0.0096 (11)	0.0132 (11)	−0.0025 (9)	−0.0010 (9)	−0.0005 (9)
C2'	0.0109 (12)	0.0105 (14)	0.0154 (13)	−0.0043 (10)	−0.0005 (10)	−0.0007 (10)
S3'	0.0144 (3)	0.0143 (4)	0.0223 (4)	0.0022 (3)	−0.0028 (3)	−0.0069 (3)
C4'	0.0177 (14)	0.0125 (15)	0.0155 (14)	−0.0033 (11)	−0.0014 (11)	−0.0022 (11)
C5'	0.0140 (13)	0.0104 (14)	0.0144 (13)	−0.0033 (11)	0.0012 (10)	−0.0029 (11)
C6'	0.0216 (15)	0.0134 (15)	0.0169 (14)	0.0019 (11)	−0.0044 (11)	−0.0028 (11)
C7'	0.0289 (16)	0.0206 (16)	0.0191 (15)	0.0032 (13)	−0.0100 (12)	−0.0056 (12)
C8'	0.0368 (18)	0.0206 (16)	0.0166 (14)	−0.0039 (14)	−0.0036 (13)	−0.0080 (12)
C9'	0.0265 (16)	0.0157 (15)	0.0213 (15)	0.0021 (12)	−0.0004 (12)	−0.0082 (12)
C10'	0.0119 (13)	0.0093 (13)	0.0170 (13)	−0.0064 (10)	−0.0013 (10)	−0.0014 (10)
C11'	0.0093 (12)	0.0095 (13)	0.0150 (13)	−0.0045 (10)	−0.0021 (10)	−0.0012 (10)
C12'	0.0143 (13)	0.0155 (15)	0.0181 (14)	−0.0043 (11)	−0.0002 (11)	−0.0023 (11)
C13'	0.0183 (14)	0.0216 (16)	0.0132 (13)	−0.0077 (12)	−0.0035 (11)	0.0007 (11)
C14'	0.0151 (14)	0.0139 (14)	0.0212 (14)	−0.0049 (11)	−0.0096 (11)	0.0019 (11)
C15'	0.0126 (13)	0.0114 (14)	0.0222 (14)	−0.0012 (11)	−0.0035 (11)	−0.0013 (11)
C97	0.0251 (16)	0.0190 (16)	0.0178 (14)	−0.0001 (13)	−0.0009 (12)	−0.0026 (12)
Cl1	0.0313 (4)	0.0276 (4)	0.0228 (4)	−0.0093 (3)	−0.0032 (3)	0.0041 (3)
Cl2	0.0270 (4)	0.0354 (5)	0.0185 (4)	−0.0021 (3)	−0.0046 (3)	−0.0058 (3)
Cl3	0.0289 (4)	0.0236 (4)	0.0264 (4)	−0.0078 (3)	−0.0048 (3)	−0.0048 (3)
C98	0.050 (2)	0.062 (3)	0.038 (2)	−0.035 (2)	−0.0144 (18)	0.0116 (19)
Cl4	0.0817 (9)	0.1328 (12)	0.0878 (9)	−0.0809 (9)	−0.0533 (7)	0.0629 (8)
Cl5	0.1107 (9)	0.0559 (7)	0.0643 (7)	−0.0447 (7)	0.0182 (6)	−0.0335 (6)
Cl6	0.0527 (6)	0.0587 (7)	0.0685 (7)	−0.0135 (5)	0.0029 (5)	−0.0381 (6)
C99	0.0222 (16)	0.0226 (17)	0.0308 (17)	−0.0056 (13)	−0.0076 (13)	0.0003 (13)
Cl7	0.0526 (6)	0.0868 (8)	0.0485 (6)	0.0014 (6)	−0.0201 (5)	−0.0343 (6)
Cl8	0.0207 (4)	0.0381 (5)	0.0544 (5)	−0.0053 (4)	−0.0109 (4)	0.0093 (4)
Cl9	0.0332 (5)	0.0363 (5)	0.0422 (5)	0.0023 (4)	0.0037 (4)	0.0035 (4)
Cl10	0.0186 (13)	0.119 (5)	0.053 (2)	−0.0228 (19)	0.0042 (13)	−0.040 (3)
Cl11	0.0549 (11)	0.0361 (10)	0.0335 (9)	−0.0055 (8)	−0.0084 (8)	−0.0106 (8)
Cl12	0.080 (3)	0.0339 (14)	0.058 (2)	−0.0109 (16)	0.0000 (19)	−0.0114 (14)

### Geometric parameters (Å, °)

**Table:**

Ir—C11	2.014 (3)	C28—C29	1.392 (3)
Ir—C11'	2.022 (2)	C28—H28	0.9500
Ir—N1'	2.060 (2)	C29—C30	1.375 (4)
Ir—N1	2.064 (2)	C29—H29	0.9500
Ir—N16	2.1264 (19)	C30—C31	1.378 (4)
Ir—N22	2.160 (2)	C30—H30	0.9500
N1—C2	1.323 (3)	C31—C32	1.390 (3)
N1—C5	1.407 (3)	C31—H31	0.9500
C2—C10	1.447 (3)	C32—H32	0.9500
C2—S3	1.720 (3)	N1'—C2'	1.322 (3)
S3—C4	1.744 (3)	N1'—C5'	1.400 (3)
C4—C9	1.398 (3)	C2'—C10'	1.453 (3)
C4—C5	1.398 (3)	C2'—S3'	1.724 (3)
C5—C6	1.399 (3)	S3'—C4'	1.742 (3)
C6—C7	1.382 (3)	C4'—C9'	1.393 (4)
C6—H6	0.9500	C4'—C5'	1.401 (3)
C7—C8	1.398 (3)	C5'—C6'	1.397 (3)
C7—H7	0.9500	C6'—C7'	1.384 (4)
C8—C9	1.377 (3)	C6'—H6'	0.9500
C8—H8	0.9500	C7'—C8'	1.394 (4)
C9—H9	0.9500	C7'—H7'	0.9500
C10—C15	1.397 (3)	C8'—C9'	1.380 (4)
C10—C11	1.412 (3)	C8'—H8'	0.9500
C11—C12	1.405 (3)	C9'—H9'	0.9500
C12—C13	1.385 (4)	C10'—C15'	1.395 (3)
C12—H12	0.9500	C10'—C11'	1.411 (3)
C13—C14	1.387 (4)	C11'—C12'	1.402 (3)
C13—H13	0.9500	C12'—C13'	1.394 (3)
C14—C15	1.379 (4)	C12'—H12'	0.9500
C14—H14	0.9500	C13'—C14'	1.388 (4)
C15—H15	0.9500	C13'—H13'	0.9500
N16—C20	1.333 (3)	C14'—C15'	1.379 (4)
N16—N17	1.365 (3)	C14'—H14'	0.9500
N17—C18	1.341 (3)	C15'—H15'	0.9500
C18—N19	1.352 (3)	C97—Cl1	1.755 (3)
C18—C27	1.486 (3)	C97—Cl2	1.759 (3)
N19—C20	1.345 (3)	C97—Cl3	1.771 (3)
C20—C21	1.447 (3)	C97—D97	1.0000
C21—N22	1.370 (3)	C98—Cl5	1.745 (4)
C21—C26	1.387 (3)	C98—Cl4	1.747 (3)
N22—C23	1.345 (3)	C98—Cl6	1.752 (4)
C23—C24	1.377 (4)	C98—D98	1.0000
C23—H23	0.9500	C99—Cl7	1.746 (3)
C24—C25	1.382 (4)	C99—Cl8	1.750 (3)
C24—H24	0.9500	C99—Cl9	1.757 (3)
C25—C26	1.387 (3)	C99—D99	1.0000
C25—H25	0.9500	C96—Cl12	1.737 (8)
C26—H26	0.9500	C96—Cl11	1.752 (6)
C27—C28	1.390 (3)	C96—Cl10	1.758 (7)
C27—C32	1.396 (4)	C96—H96	1.0000
			
C11—Ir—C11'	88.18 (9)	C21—C26—H26	120.3
C11—Ir—N1'	94.17 (9)	C28—C27—C32	119.5 (2)
C11'—Ir—N1'	79.98 (9)	C28—C27—C18	120.0 (2)
C11—Ir—N1	79.54 (9)	C32—C27—C18	120.4 (2)
C11'—Ir—N1	92.98 (9)	C27—C28—C29	120.1 (3)
N1'—Ir—N1	170.77 (8)	C27—C28—H28	120.0
C11—Ir—N16	102.78 (8)	C29—C28—H28	120.0
C11'—Ir—N16	168.90 (8)	C30—C29—C28	120.0 (3)
N1'—Ir—N16	97.25 (8)	C30—C29—H29	120.0
N1—Ir—N16	90.77 (8)	C28—C29—H29	120.0
C11—Ir—N22	178.49 (9)	C29—C30—C31	120.4 (2)
C11'—Ir—N22	93.05 (8)	C29—C30—H30	119.8
N1'—Ir—N22	86.91 (8)	C31—C30—H30	119.8
N1—Ir—N22	99.51 (8)	C30—C31—C32	120.3 (3)
N16—Ir—N22	76.03 (8)	C30—C31—H31	119.9
C2—N1—C5	110.8 (2)	C32—C31—H31	119.9
C2—N1—Ir	114.19 (17)	C31—C32—C27	119.7 (3)
C5—N1—Ir	134.97 (17)	C31—C32—H32	120.1
N1—C2—C10	117.8 (2)	C27—C32—H32	120.1
N1—C2—S3	115.97 (19)	C2'—N1'—C5'	111.6 (2)
C10—C2—S3	126.14 (19)	C2'—N1'—Ir	113.96 (16)
C2—S3—C4	89.36 (12)	C5'—N1'—Ir	134.48 (16)
C9—C4—C5	121.9 (2)	N1'—C2'—C10'	117.8 (2)
C9—C4—S3	127.7 (2)	N1'—C2'—S3'	115.30 (18)
C5—C4—S3	110.37 (19)	C10'—C2'—S3'	126.80 (19)
C4—C5—C6	119.5 (2)	C2'—S3'—C4'	89.47 (12)
C4—C5—N1	113.5 (2)	C9'—C4'—C5'	121.9 (2)
C6—C5—N1	127.1 (2)	C9'—C4'—S3'	127.6 (2)
C7—C6—C5	118.6 (2)	C5'—C4'—S3'	110.33 (19)
C7—C6—H6	120.7	C6'—C5'—N1'	127.0 (2)
C5—C6—H6	120.7	C6'—C5'—C4'	119.7 (2)
C6—C7—C8	121.3 (2)	N1'—C5'—C4'	113.2 (2)
C6—C7—H7	119.3	C7'—C6'—C5'	118.2 (2)
C8—C7—H7	119.3	C7'—C6'—H6'	120.9
C9—C8—C7	121.0 (2)	C5'—C6'—H6'	120.9
C9—C8—H8	119.5	C6'—C7'—C8'	121.6 (2)
C7—C8—H8	119.5	C6'—C7'—H7'	119.2
C8—C9—C4	117.7 (2)	C8'—C7'—H7'	119.2
C8—C9—H9	121.1	C9'—C8'—C7'	121.0 (3)
C4—C9—H9	121.1	C9'—C8'—H8'	119.5
C15—C10—C11	122.7 (2)	C7'—C8'—H8'	119.5
C15—C10—C2	124.8 (2)	C8'—C9'—C4'	117.6 (3)
C11—C10—C2	112.4 (2)	C8'—C9'—H9'	121.2
C12—C11—C10	116.1 (2)	C4'—C9'—H9'	121.2
C12—C11—Ir	128.7 (2)	C15'—C10'—C11'	123.4 (2)
C10—C11—Ir	115.08 (18)	C15'—C10'—C2'	123.5 (2)
C13—C12—C11	120.9 (2)	C11'—C10'—C2'	113.0 (2)
C13—C12—H12	119.6	C12'—C11'—C10'	115.6 (2)
C11—C12—H12	119.6	C12'—C11'—Ir	129.8 (2)
C12—C13—C14	121.8 (3)	C10'—C11'—Ir	114.42 (17)
C12—C13—H13	119.1	C13'—C12'—C11'	121.2 (3)
C14—C13—H13	119.1	C13'—C12'—H12'	119.4
C15—C14—C13	119.0 (2)	C11'—C12'—H12'	119.4
C15—C14—H14	120.5	C14'—C13'—C12'	121.3 (2)
C13—C14—H14	120.5	C14'—C13'—H13'	119.3
C14—C15—C10	119.4 (2)	C12'—C13'—H13'	119.3
C14—C15—H15	120.3	C15'—C14'—C13'	119.3 (2)
C10—C15—H15	120.3	C15'—C14'—H14'	120.3
C20—N16—N17	107.33 (19)	C13'—C14'—H14'	120.3
C20—N16—Ir	115.60 (15)	C14'—C15'—C10'	119.0 (2)
N17—N16—Ir	137.07 (16)	C14'—C15'—H15'	120.5
C18—N17—N16	103.6 (2)	C10'—C15'—H15'	120.5
N17—C18—N19	114.9 (2)	Cl1—C97—Cl2	111.48 (16)
N17—C18—C27	121.6 (2)	Cl1—C97—Cl3	109.43 (14)
N19—C18—C27	123.5 (2)	Cl2—C97—Cl3	110.84 (15)
C20—N19—C18	100.8 (2)	Cl1—C97—D97	108.3
N16—C20—N19	113.2 (2)	Cl2—C97—D97	108.3
N16—C20—C21	118.9 (2)	Cl3—C97—D97	108.3
N19—C20—C21	127.9 (2)	Cl5—C98—Cl4	110.2 (2)
N22—C21—C26	121.2 (2)	Cl5—C98—Cl6	110.47 (18)
N22—C21—C20	113.6 (2)	Cl4—C98—Cl6	111.8 (2)
C26—C21—C20	125.2 (2)	Cl5—C98—D98	108.1
C23—N22—C21	118.4 (2)	Cl4—C98—D98	108.1
C23—N22—Ir	125.73 (17)	Cl6—C98—D98	108.1
C21—N22—Ir	115.91 (16)	Cl7—C99—Cl8	110.99 (17)
N22—C23—C24	122.7 (2)	Cl7—C99—Cl9	110.85 (15)
N22—C23—H23	118.6	Cl8—C99—Cl9	111.02 (15)
C24—C23—H23	118.6	Cl7—C99—D99	107.9
C23—C24—C25	119.2 (2)	Cl8—C99—D99	107.9
C23—C24—H24	120.4	Cl9—C99—D99	107.9
C25—C24—H24	120.4	Cl12—C96—Cl11	110.5 (4)
C24—C25—C26	119.1 (3)	Cl12—C96—Cl10	111.1 (4)
C24—C25—H25	120.4	Cl11—C96—Cl10	109.3 (4)
C26—C25—H25	120.4	Cl12—C96—H96	108.6
C25—C26—C21	119.4 (2)	Cl11—C96—H96	108.6
C25—C26—H26	120.3	Cl10—C96—H96	108.6
			
C11—Ir—N1—C2	−6.33 (16)	C11'—Ir—N22—C23	2.0 (2)
C11'—Ir—N1—C2	81.27 (17)	N1'—Ir—N22—C23	81.8 (2)
N16—Ir—N1—C2	−109.18 (16)	N1—Ir—N22—C23	−91.5 (2)
N22—Ir—N1—C2	174.86 (16)	N16—Ir—N22—C23	−179.9 (2)
C11—Ir—N1—C5	173.0 (2)	C11'—Ir—N22—C21	−178.67 (18)
C11'—Ir—N1—C5	−99.5 (2)	N1'—Ir—N22—C21	−98.88 (18)
N16—Ir—N1—C5	70.1 (2)	N1—Ir—N22—C21	87.79 (18)
N22—Ir—N1—C5	−5.9 (2)	N16—Ir—N22—C21	−0.63 (17)
C5—N1—C2—C10	−176.80 (19)	C21—N22—C23—C24	−1.3 (4)
Ir—N1—C2—C10	2.7 (3)	Ir—N22—C23—C24	178.0 (2)
C5—N1—C2—S3	0.3 (2)	N22—C23—C24—C25	0.1 (4)
Ir—N1—C2—S3	179.74 (10)	C23—C24—C25—C26	1.2 (4)
N1—C2—S3—C4	−0.48 (18)	C24—C25—C26—C21	−1.3 (4)
C10—C2—S3—C4	176.3 (2)	N22—C21—C26—C25	0.1 (4)
C2—S3—C4—C9	−179.1 (2)	C20—C21—C26—C25	−177.9 (3)
C2—S3—C4—C5	0.53 (18)	N17—C18—C27—C28	170.1 (2)
C9—C4—C5—C6	−0.4 (3)	N19—C18—C27—C28	−8.5 (4)
S3—C4—C5—C6	179.93 (17)	N17—C18—C27—C32	−6.5 (4)
C9—C4—C5—N1	179.1 (2)	N19—C18—C27—C32	174.9 (2)
S3—C4—C5—N1	−0.5 (2)	C32—C27—C28—C29	1.8 (4)
C2—N1—C5—C4	0.2 (3)	C18—C27—C28—C29	−174.8 (2)
Ir—N1—C5—C4	−179.15 (16)	C27—C28—C29—C30	−0.3 (4)
C2—N1—C5—C6	179.7 (2)	C28—C29—C30—C31	−1.3 (4)
Ir—N1—C5—C6	0.4 (4)	C29—C30—C31—C32	1.4 (4)
C4—C5—C6—C7	0.2 (3)	C30—C31—C32—C27	0.1 (4)
N1—C5—C6—C7	−179.3 (2)	C28—C27—C32—C31	−1.7 (4)
C5—C6—C7—C8	−0.1 (4)	C18—C27—C32—C31	174.9 (2)
C6—C7—C8—C9	0.1 (4)	C11—Ir—N1'—C2'	95.28 (18)
C7—C8—C9—C4	−0.3 (3)	C11'—Ir—N1'—C2'	7.87 (17)
C5—C4—C9—C8	0.5 (3)	N16—Ir—N1'—C2'	−161.26 (17)
S3—C4—C9—C8	−179.98 (18)	N22—Ir—N1'—C2'	−85.78 (17)
N1—C2—C10—C15	−178.6 (2)	C11—Ir—N1'—C5'	−84.3 (2)
S3—C2—C10—C15	4.7 (3)	C11'—Ir—N1'—C5'	−171.7 (2)
N1—C2—C10—C11	4.8 (3)	N16—Ir—N1'—C5'	19.2 (2)
S3—C2—C10—C11	−171.96 (17)	N22—Ir—N1'—C5'	94.6 (2)
C15—C10—C11—C12	−3.5 (3)	C5'—N1'—C2'—C10'	173.0 (2)
C2—C10—C11—C12	173.2 (2)	Ir—N1'—C2'—C10'	−6.6 (3)
C15—C10—C11—Ir	173.17 (18)	C5'—N1'—C2'—S3'	−3.8 (3)
C2—C10—C11—Ir	−10.1 (3)	Ir—N1'—C2'—S3'	176.50 (11)
C11'—Ir—C11—C12	91.8 (2)	N1'—C2'—S3'—C4'	2.0 (2)
N1'—Ir—C11—C12	12.0 (2)	C10'—C2'—S3'—C4'	−174.5 (2)
N1—Ir—C11—C12	−174.8 (2)	C2'—S3'—C4'—C9'	175.7 (3)
N16—Ir—C11—C12	−86.4 (2)	C2'—S3'—C4'—C5'	0.3 (2)
C11'—Ir—C11—C10	−84.38 (18)	C2'—N1'—C5'—C6'	−172.2 (2)
N1'—Ir—C11—C10	−164.20 (17)	Ir—N1'—C5'—C6'	7.4 (4)
N1—Ir—C11—C10	8.99 (17)	C2'—N1'—C5'—C4'	4.0 (3)
N16—Ir—C11—C10	97.39 (18)	Ir—N1'—C5'—C4'	−176.37 (18)
C10—C11—C12—C13	3.3 (3)	C9'—C4'—C5'—C6'	−1.7 (4)
Ir—C11—C12—C13	−172.88 (18)	S3'—C4'—C5'—C6'	174.0 (2)
C11—C12—C13—C14	−0.7 (4)	C9'—C4'—C5'—N1'	−178.2 (2)
C12—C13—C14—C15	−1.9 (4)	S3'—C4'—C5'—N1'	−2.5 (3)
C13—C14—C15—C10	1.7 (4)	N1'—C5'—C6'—C7'	176.5 (3)
C11—C10—C15—C14	1.1 (4)	C4'—C5'—C6'—C7'	0.5 (4)
C2—C10—C15—C14	−175.2 (2)	C5'—C6'—C7'—C8'	0.7 (4)
C11—Ir—N16—C20	−177.88 (18)	C6'—C7'—C8'—C9'	−0.6 (4)
N1'—Ir—N16—C20	86.15 (18)	C7'—C8'—C9'—C4'	−0.5 (4)
N1—Ir—N16—C20	−98.43 (18)	C5'—C4'—C9'—C8'	1.7 (4)
N22—Ir—N16—C20	1.18 (18)	S3'—C4'—C9'—C8'	−173.2 (2)
C11—Ir—N16—N17	1.8 (2)	N1'—C2'—C10'—C15'	−177.1 (2)
N1'—Ir—N16—N17	−94.2 (2)	S3'—C2'—C10'—C15'	−0.6 (4)
N1—Ir—N16—N17	81.2 (2)	N1'—C2'—C10'—C11'	0.1 (3)
N22—Ir—N16—N17	−179.2 (2)	S3'—C2'—C10'—C11'	176.57 (18)
C20—N16—N17—C18	0.3 (3)	C15'—C10'—C11'—C12'	0.2 (4)
Ir—N16—N17—C18	−179.34 (19)	C2'—C10'—C11'—C12'	−177.0 (2)
N16—N17—C18—N19	−0.1 (3)	C15'—C10'—C11'—Ir	−176.18 (19)
N16—N17—C18—C27	−178.8 (2)	C2'—C10'—C11'—Ir	6.6 (3)
N17—C18—N19—C20	−0.2 (3)	C11—Ir—C11'—C12'	81.9 (2)
C27—C18—N19—C20	178.5 (2)	N1'—Ir—C11'—C12'	176.5 (2)
N17—N16—C20—N19	−0.5 (3)	N1—Ir—C11'—C12'	2.5 (2)
Ir—N16—C20—N19	179.27 (16)	N22—Ir—C11'—C12'	−97.2 (2)
N17—N16—C20—C21	178.6 (2)	C11—Ir—C11'—C10'	−102.35 (19)
Ir—N16—C20—C21	−1.6 (3)	N1'—Ir—C11'—C10'	−7.78 (17)
C18—N19—C20—N16	0.4 (3)	N1—Ir—C11'—C10'	178.23 (18)
C18—N19—C20—C21	−178.6 (3)	N22—Ir—C11'—C10'	78.53 (18)
N16—C20—C21—N22	1.0 (3)	C10'—C11'—C12'—C13'	1.1 (4)
N19—C20—C21—N22	−180.0 (2)	Ir—C11'—C12'—C13'	176.80 (19)
N16—C20—C21—C26	179.2 (2)	C11'—C12'—C13'—C14'	−1.3 (4)
N19—C20—C21—C26	−1.8 (4)	C12'—C13'—C14'—C15'	0.1 (4)
C26—C21—N22—C23	1.2 (4)	C13'—C14'—C15'—C10'	1.1 (4)
C20—C21—N22—C23	179.4 (2)	C11'—C10'—C15'—C14'	−1.3 (4)
C26—C21—N22—Ir	−178.2 (2)	C2'—C10'—C15'—C14'	175.6 (2)
C20—C21—N22—Ir	0.0 (3)		

### Hydrogen-bond geometry (Å, °)

**Table:**

Cg is the centroid of the C27–C32 ring.

**Table:**

*D*—H···*A*	*D*—H	H···*A*	*D*···*A*	*D*—H···*A*
C99—D99···N19	1.00	2.18	3.180 (4)	175
C98—D98···S3'	1.00	3.04	3.660 (3)	121
C97—D97···Cg	1.00	2.50	3.50	173
